# Bis(2-amino­pyrimidine-κ*N* ^1^)diaqua­dinitrato-κ*O*;κ^2^ *O*,*O*′-cadmium(II) monohydrate

**DOI:** 10.1107/S1600536808006521

**Published:** 2008-03-14

**Authors:** Xi-Shi Tai, Yi-Min Feng, Lin-Tong Wang

**Affiliations:** a Department of Chemistry and Chemical Engineering, Weifang University, Weifang 261061, People’s Republic of China

## Abstract

In the title compound, [Cd(NO_3_)_2_(C_4_H_5_N_3_)_2_(H_2_O)_2_]·H_2_O, the Cd atom is seven-coordinated by two 2-amino­pyrimidine mol­ecules, two water mol­ecules, one bidentate nitrate anion and one monodentate nitrate anion. A network of N—H⋯O, N—H⋯N and O—H⋯O hydrogen bonds helps to consolidate the crystal structure.

## Related literature

For related literature, see: Cui *et al.* (2003 ▶).
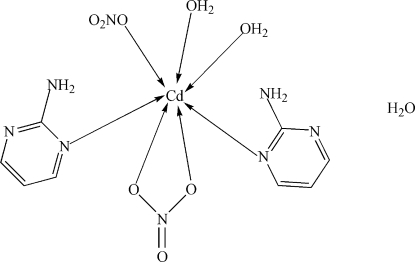


## Experimental

### 

#### Crystal data

[Cd(NO_3_)_2_(C_4_H_5_N_3_)_2_(H_2_O)_2_]·H_2_O
*M* *_r_* = 480.69
Monoclinic, 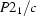

*a* = 13.451 (2) Å
*b* = 7.8692 (14) Å
*c* = 16.699 (3) Å
β = 101.330 (2)°
*V* = 1733.2 (5) Å^3^
*Z* = 4
Mo *K*α radiation
μ = 1.32 mm^−1^
*T* = 298 (2) K
0.57 × 0.47 × 0.34 mm

#### Data collection

Bruker SMART CCD diffractometer
Absorption correction: multi-scan (*SADABS*; Bruker, 2000 ▶) *T*_min_ = 0.519, *T*_max_ = 0.662
9748 measured reflections
3771 independent reflections
3209 reflections with *I* > 2σ(*I*)
*R*_int_ = 0.046

#### Refinement

*R*[*F* ^2^ > 2σ(*F* ^2^)] = 0.029
*wR*(*F* ^2^) = 0.079
*S* = 1.04
3771 reflections
236 parameters
H-atom parameters constrained
Δρ_max_ = 0.83 e Å^−3^
Δρ_min_ = −0.99 e Å^−3^

### 

Data collection: *SMART* (Bruker, 2000 ▶); cell refinement: *SAINT* (Bruker, 2000 ▶); data reduction: *SAINT*; program(s) used to solve structure: *SHELXS97* (Sheldrick, 2008 ▶); program(s) used to refine structure: *SHELXL97* (Sheldrick, 2008 ▶); molecular graphics: *SHELXTL* (Sheldrick, 2008 ▶); software used to prepare material for publication: *SHELXTL*.

## Supplementary Material

Crystal structure: contains datablocks global, I. DOI: 10.1107/S1600536808006521/hb2693sup1.cif

Structure factors: contains datablocks I. DOI: 10.1107/S1600536808006521/hb2693Isup2.hkl

Additional supplementary materials:  crystallographic information; 3D view; checkCIF report

## Figures and Tables

**Table 1:** Selected bond lengths (Å)

Cd1—O7	2.3009 (19)
Cd1—O8	2.335 (2)
Cd1—N1	2.361 (3)
Cd1—N4	2.399 (3)
Cd1—O4	2.407 (2)
Cd1—O2	2.512 (2)
Cd1—O1	2.640 (3)

**Table 2:** Hydrogen-bond geometry (Å, °)

*D*—H⋯*A*	*D*—H	H⋯*A*	*D*⋯*A*	*D*—H⋯*A*
N3—H3*A*⋯O5^i^	0.86	2.29	3.105 (4)	158
N3—H3*B*⋯O7	0.86	2.10	2.945 (4)	167
N6—H6*A*⋯N5^ii^	0.86	2.20	3.054 (4)	170
N6—H6*B*⋯O2	0.86	2.19	2.931 (4)	144
N6—H6*B*⋯O3^iii^	0.86	2.52	3.171 (4)	133
O7—H7*A*⋯O9^iv^	0.85	1.94	2.787 (3)	178
O7—H7*B*⋯O9^v^	0.85	1.87	2.724 (3)	178
O8—H8*A*⋯O3^vi^	0.85	1.97	2.820 (3)	176
O8—H8*B*⋯O3^iii^	0.85	2.09	2.936 (3)	176
O9—H9*A*⋯O5^iv^	0.85	2.44	3.255 (3)	162
O9—H9*A*⋯O7^iv^	0.85	2.28	2.787 (3)	119
O9—H9*B*⋯O6^vii^	0.85	1.99	2.809 (4)	161

Symmetry codes: (i) 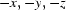; (ii) 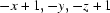; (iii) 
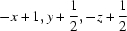
; (iv) 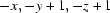; (v) 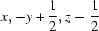; (vi) 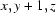; (vii) 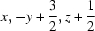.

**Fig. 1.**
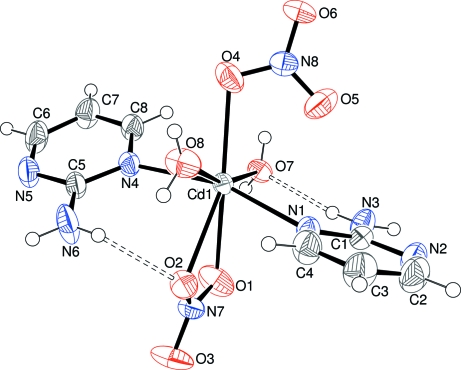
The molecular structure of the complex ion in (I) showing 50% displacement ellipsoids for the non-hydrogen atoms. Hydrogen bonds are indicated by double-dashed lines.

## supplementary crystallographic information

### Comment

As part of the ongoing studies (Cui *et al.*, 2003) of the coordination chemistry of Cd(II) ion, we now report the synthesis and structure of the title compound, (I), (Fig. 1).

The Cd atom in (I) is seven-coordinate with two N-donor 2-aminopyrimidine molecules, two water molecules and one bidentate NO_3_^-^ and one monodentate NO_3_^-^ ions (Table 1). The coordination polyhedron around Cd is a distorted pengonal bipyramidal with the N atoms in the axial positions [N1—Cd1—N4 = 164.13 (9)°]. The dihedral angle between the aromatic ring planes is 33.76 (17)°.

A network of N—H···O, N—H···N and O—H···O hydrogen bonds (Table 2) helps to establish the structure of (I).

### Experimental

A solution of 0.5 mmol C d(NO_3_)_2_.4H_2_O in 10 ml 95% ethanol was added to a solution of 1.0 mmol 2-aminopyrimidine in 10 ml e thanol at room temperature. The mixture was refluxed for 2 h with stirring, then the resulting precipitate was filtered, washed, and dried *in vacuo* over P_4_O_10_ for 48 h. Colourless blocks of (I) were recrystallized from methanol at room temperature.

### Refinement

The H atoms were placed geometrically (C—H = 0.93–0.96 Å, O—H = 0.82 Å, N—H = 0.86 Å) and refined as riding with *U*_iso_(H) = 1.2*U*_eq_(carrier) or 1.5*U*_eq_(methyl C). Some short H···H contacts arise from this geometrical placement scheme and the positions of the water H atoms should be regarded as less certain.

### Crystal data

**Table:**

[Cd(NO_3_)_2_(C_4_H_5_N_3_)_2_(H_2_O)_2_]·H_2_O	*F*_000_ = 960
*M**_r_* = 480.69	*D*_x_ = 1.842 Mg m^−^^3^
Monoclinic, *P*2_1_/*c*	Mo *K*α radiation λ = 0.71073 Å
Hall symbol: -P 2ybc	Cell parameters from 6206 reflections
*a* = 13.451 (2) Å	θ = 2.6–28.2º
*b* = 7.8692 (14) Å	µ = 1.32 mm^−^^1^
*c* = 16.699 (3) Å	*T* = 298 (2) K
β = 101.330 (2)º	Block, colourless
*V* = 1733.2 (5) Å^3^	0.57 × 0.47 × 0.34 mm
*Z* = 4	

### Data collection

**Table:**

Bruker SMART CCD diffractometer	3771 independent reflections
Radiation source: fine-focus sealed tube	3209 reflections with *I* > 2σ(*I*)
Monochromator: graphite	*R*_int_ = 0.046
*T* = 298(2) K	θ_max_ = 27.0º
ω scans	θ_min_ = 1.5º
Absorption correction: multi-scan(SADABS; Bruker, 2000)	*h* = −16→17
*T*_min_ = 0.519, *T*_max_ = 0.662	*k* = −10→9
9748 measured reflections	*l* = −21→17

### Refinement

**Table:**

Refinement on *F*^2^	Hydrogen site location: inferred from neighbouring sites
Least-squares matrix: full	H-atom parameters constrained
*R*[*F*^2^ > 2σ(*F*^2^)] = 0.030	*w* = 1/[σ^2^(*F*_o_^2^) + (0.0376*P*)^2^ + 0.7065*P*] where *P* = (*F*_o_^2^ + 2*F*_c_^2^)/3
*wR*(*F*^2^) = 0.079	(Δ/σ)_max_ = 0.001
*S* = 1.04	Δρ_max_ = 0.83 e Å^−^^3^
3771 reflections	Δρ_min_ = −0.99 e Å^−^^3^
236 parameters	Extinction correction: SHELXL97 (Sheldrick, 2008), Fc^*^=kFc[1+0.001xFc^2^λ^3^/sin(2θ)]^-1/4^
Primary atom site location: structure-invariant direct methods	Extinction coefficient: 0.0486 (12)
Secondary atom site location: difference Fourier map	

### Special details

**Table:**

Geometry. All e.s.d.'s (except the e.s.d. in the dihedral angle between two l.s. planes) are estimated using the full covariance matrix. The cell e.s.d.'s are taken into account individually in the estimation of e.s.d.'s in distances, angles and torsion angles; correlations between e.s.d.'s in cell parameters are only used when they are defined by crystal symmetry. An approximate (isotropic) treatment of cell e.s.d.'s is used for estimating e.s.d.'s involving l.s. planes.
Refinement. Refinement of *F*^2^ against ALL reflections. The weighted *R*-factor *wR* and goodness of fit *S* are based on *F*^2^, conventional *R*-factors *R* are based on *F*, with *F* set to zero for negative *F*^2^. The threshold expression of *F*^2^ > σ(*F*^2^) is used only for calculating *R*-factors(gt) *etc*. and is not relevant to the choice of reflections for refinement. *R*-factors based on *F*^2^ are statistically about twice as large as those based on *F*, and *R*- factors based on ALL data will be even larger.

### Fractional atomic coordinates and isotropic or equivalent isotropic displacement parameters (Å^2^)

**Table:**

	*x*	*y*	*z*	*U*_iso_*/*U*_eq_	
Cd1	0.247663 (13)	0.03671 (3)	0.205049 (12)	0.02590 (11)	
N1	0.26466 (19)	−0.0114 (4)	0.06883 (17)	0.0349 (6)	
N2	0.2023 (2)	−0.0805 (4)	−0.07156 (17)	0.0471 (7)	
N3	0.10954 (19)	−0.1429 (4)	0.02583 (17)	0.0441 (7)	
H3A	0.0637	−0.1851	−0.0122	0.053*	
H3B	0.1010	−0.1433	0.0755	0.053*	
N4	0.26970 (18)	0.0332 (3)	0.35116 (16)	0.0326 (6)	
N5	0.3562 (2)	0.0073 (4)	0.49009 (17)	0.0452 (7)	
N6	0.4438 (2)	−0.0069 (5)	0.38605 (19)	0.0649 (11)	
H6A	0.4983	−0.0206	0.4223	0.078*	
H6B	0.4468	−0.0050	0.3351	0.078*	
N7	0.36446 (17)	−0.2853 (3)	0.22553 (16)	0.0343 (6)	
N8	0.10409 (17)	0.3297 (3)	0.14881 (17)	0.0349 (6)	
O1	0.27101 (15)	−0.2961 (4)	0.21618 (17)	0.0569 (7)	
O2	0.40409 (16)	−0.1442 (3)	0.21968 (15)	0.0416 (5)	
O3	0.41920 (17)	−0.4123 (3)	0.2418 (2)	0.0610 (8)	
O4	0.16425 (16)	0.3041 (3)	0.21572 (16)	0.0496 (6)	
O5	0.0921 (2)	0.2158 (3)	0.09573 (16)	0.0580 (7)	
O6	0.0575 (2)	0.4627 (3)	0.13678 (18)	0.0554 (7)	
O7	0.09014 (14)	−0.0842 (3)	0.19628 (13)	0.0323 (5)	
H7A	0.0430	−0.0155	0.2014	0.039*	
H7B	0.0838	−0.1732	0.2236	0.039*	
O8	0.37736 (15)	0.2381 (3)	0.21821 (16)	0.0471 (6)	
H8A	0.3866	0.3445	0.2246	0.056*	
H8B	0.4348	0.1893	0.2293	0.056*	
O9	0.06716 (14)	0.8659 (3)	0.78566 (13)	0.0373 (5)	
H9A	0.0160	0.8574	0.8083	0.045*	
H9B	0.0501	0.9217	0.7415	0.045*	
C1	0.1937 (2)	−0.0771 (4)	0.00777 (19)	0.0346 (7)	
C2	0.2866 (3)	−0.0177 (5)	−0.0896 (2)	0.0549 (10)	
H2	0.2937	−0.0173	−0.1439	0.066*	
C3	0.3639 (3)	0.0467 (5)	−0.0314 (3)	0.0543 (10)	
H3	0.4234	0.0880	−0.0448	0.065*	
C4	0.3488 (2)	0.0470 (5)	0.0473 (2)	0.0473 (9)	
H4	0.3999	0.0902	0.0880	0.057*	
C5	0.3540 (2)	0.0118 (4)	0.40915 (19)	0.0347 (7)	
C6	0.2691 (3)	0.0261 (5)	0.5134 (2)	0.0506 (9)	
H6	0.2684	0.0218	0.5690	0.061*	
C7	0.1779 (2)	0.0521 (5)	0.4590 (2)	0.0476 (9)	
H7	0.1172	0.0670	0.4768	0.057*	
C8	0.1826 (2)	0.0548 (4)	0.3780 (2)	0.0390 (8)	
H8	0.1231	0.0721	0.3399	0.047*	

### Atomic displacement parameters (Å^2^)

**Table:**

	*U*^11^	*U*^22^	*U*^33^	*U*^12^	*U*^13^	*U*^23^
Cd1	0.02132 (13)	0.02807 (15)	0.02691 (14)	0.00027 (7)	0.00130 (8)	−0.00120 (8)
N1	0.0284 (12)	0.0439 (15)	0.0309 (14)	−0.0010 (11)	0.0027 (10)	−0.0027 (12)
N2	0.0498 (16)	0.0603 (19)	0.0316 (15)	−0.0003 (14)	0.0091 (12)	−0.0078 (15)
N3	0.0411 (14)	0.0565 (19)	0.0344 (14)	−0.0135 (13)	0.0068 (11)	−0.0118 (14)
N4	0.0259 (11)	0.0400 (15)	0.0300 (13)	0.0013 (10)	0.0007 (10)	−0.0027 (11)
N5	0.0373 (14)	0.068 (2)	0.0273 (14)	0.0051 (13)	−0.0008 (11)	−0.0018 (14)
N6	0.0285 (14)	0.135 (3)	0.0289 (15)	0.0179 (16)	−0.0006 (12)	0.0009 (18)
N7	0.0295 (12)	0.0305 (14)	0.0426 (15)	0.0048 (10)	0.0067 (10)	−0.0004 (12)
N8	0.0287 (11)	0.0337 (14)	0.0438 (15)	0.0006 (11)	0.0109 (11)	0.0013 (12)
O1	0.0241 (10)	0.0641 (18)	0.083 (2)	0.0028 (11)	0.0109 (11)	0.0149 (15)
O2	0.0442 (11)	0.0270 (12)	0.0487 (14)	−0.0064 (9)	−0.0025 (10)	0.0000 (10)
O3	0.0417 (13)	0.0315 (13)	0.112 (3)	0.0132 (11)	0.0204 (14)	0.0121 (15)
O4	0.0369 (11)	0.0470 (14)	0.0571 (15)	0.0023 (10)	−0.0096 (10)	0.0069 (12)
O5	0.0936 (19)	0.0413 (14)	0.0451 (15)	−0.0025 (13)	0.0288 (14)	−0.0077 (12)
O6	0.0562 (15)	0.0451 (15)	0.0617 (18)	0.0282 (12)	0.0039 (13)	0.0021 (13)
O7	0.0255 (9)	0.0335 (11)	0.0384 (11)	0.0016 (8)	0.0069 (8)	0.0045 (10)
O8	0.0296 (10)	0.0326 (12)	0.0763 (18)	−0.0078 (9)	0.0038 (10)	−0.0030 (12)
O9	0.0358 (10)	0.0433 (13)	0.0343 (11)	0.0034 (9)	0.0103 (9)	0.0027 (10)
C1	0.0370 (15)	0.0350 (17)	0.0305 (15)	0.0058 (12)	0.0033 (12)	−0.0055 (13)
C2	0.059 (2)	0.073 (3)	0.0362 (19)	0.0019 (19)	0.0197 (17)	−0.0019 (19)
C3	0.0426 (19)	0.076 (3)	0.048 (2)	−0.0033 (17)	0.0194 (16)	−0.001 (2)
C4	0.0312 (16)	0.066 (3)	0.044 (2)	−0.0030 (15)	0.0043 (14)	−0.0048 (18)
C5	0.0278 (14)	0.0475 (18)	0.0265 (15)	0.0022 (13)	−0.0005 (12)	−0.0014 (14)
C6	0.050 (2)	0.076 (3)	0.0254 (16)	−0.0004 (18)	0.0075 (15)	−0.0066 (17)
C7	0.0344 (16)	0.072 (3)	0.0373 (18)	−0.0029 (15)	0.0101 (14)	−0.0116 (18)
C8	0.0235 (14)	0.055 (2)	0.0368 (17)	0.0013 (13)	0.0023 (12)	−0.0070 (15)

### Geometric parameters (Å, °)

**Table:**

Cd1—O7	2.3009 (19)	N7—O3	1.239 (3)
Cd1—O8	2.335 (2)	N7—O2	1.244 (3)
Cd1—N1	2.361 (3)	N8—O6	1.216 (3)
Cd1—N4	2.399 (3)	N8—O5	1.249 (3)
Cd1—O4	2.407 (2)	N8—O4	1.260 (3)
Cd1—O2	2.512 (2)	O7—H7A	0.8500
Cd1—O1	2.640 (3)	O7—H7B	0.8500
N1—C4	1.335 (4)	O8—H8A	0.8500
N1—C1	1.355 (4)	O8—H8B	0.8501
N2—C2	1.324 (5)	O9—H9A	0.8500
N2—C1	1.353 (4)	O9—H9B	0.8500
N3—C1	1.332 (4)	C2—C3	1.373 (6)
N3—H3A	0.8600	C2—H2	0.9300
N3—H3B	0.8600	C3—C4	1.370 (5)
N4—C8	1.345 (4)	C3—H3	0.9300
N4—C5	1.349 (4)	C4—H4	0.9300
N5—C6	1.315 (4)	C6—C7	1.390 (5)
N5—C5	1.346 (4)	C6—H6	0.9300
N6—C5	1.347 (4)	C7—C8	1.366 (5)
N6—H6A	0.8600	C7—H7	0.9300
N6—H6B	0.8600	C8—H8	0.9300
N7—O1	1.239 (3)		
			
O7—Cd1—O8	161.47 (8)	O6—N8—O5	120.7 (3)
O7—Cd1—N1	97.74 (8)	O6—N8—O4	120.3 (3)
O8—Cd1—N1	89.28 (9)	O5—N8—O4	119.0 (3)
O7—Cd1—N4	89.33 (8)	N7—O1—Cd1	92.53 (19)
O8—Cd1—N4	88.35 (9)	N7—O2—Cd1	98.61 (16)
N1—Cd1—N4	164.13 (9)	N8—O4—Cd1	107.6 (2)
O7—Cd1—O4	85.94 (8)	Cd1—O7—H7A	115.3
O8—Cd1—O4	75.54 (8)	Cd1—O7—H7B	119.7
N1—Cd1—O4	110.25 (9)	H7A—O7—H7B	108.3
N4—Cd1—O4	84.32 (9)	Cd1—O8—H8A	140.2
O7—Cd1—O2	121.01 (7)	Cd1—O8—H8B	110.1
O8—Cd1—O2	77.26 (8)	H8A—O8—H8B	108.3
N1—Cd1—O2	76.42 (8)	H9A—O9—H9B	108.8
N4—Cd1—O2	87.76 (8)	N3—C1—N2	117.0 (3)
O4—Cd1—O2	151.84 (7)	N3—C1—N1	118.8 (3)
O7—Cd1—O1	71.92 (7)	N2—C1—N1	124.2 (3)
O8—Cd1—O1	126.19 (7)	N2—C2—C3	122.7 (4)
N1—Cd1—O1	82.89 (9)	N2—C2—H2	118.6
N4—Cd1—O1	85.87 (9)	C3—C2—H2	118.6
O4—Cd1—O1	155.85 (7)	C4—C3—C2	116.4 (3)
O2—Cd1—O1	49.10 (6)	C4—C3—H3	121.8
C4—N1—C1	116.0 (3)	C2—C3—H3	121.8
C4—N1—Cd1	116.9 (2)	N1—C4—C3	123.4 (3)
C1—N1—Cd1	126.8 (2)	N1—C4—H4	118.3
C2—N2—C1	117.2 (3)	C3—C4—H4	118.3
C1—N3—H3A	120.0	N5—C5—N6	116.1 (3)
C1—N3—H3B	120.0	N5—C5—N4	125.0 (3)
H3A—N3—H3B	120.0	N6—C5—N4	118.9 (3)
C8—N4—C5	116.1 (3)	N5—C6—C7	123.1 (3)
C8—N4—Cd1	113.3 (2)	N5—C6—H6	118.4
C5—N4—Cd1	130.5 (2)	C7—C6—H6	118.4
C6—N5—C5	116.7 (3)	C8—C7—C6	116.3 (3)
C5—N6—H6A	120.0	C8—C7—H7	121.9
C5—N6—H6B	120.0	C6—C7—H7	121.9
H6A—N6—H6B	120.0	N4—C8—C7	122.8 (3)
O1—N7—O3	121.1 (3)	N4—C8—H8	118.6
O1—N7—O2	119.4 (3)	C7—C8—H8	118.6
O3—N7—O2	119.5 (2)		
			
O7—Cd1—N1—C4	178.2 (2)	O8—Cd1—O2—N7	172.1 (2)
O8—Cd1—N1—C4	15.4 (3)	N1—Cd1—O2—N7	−95.49 (19)
N4—Cd1—N1—C4	−66.0 (5)	N4—Cd1—O2—N7	83.32 (19)
O4—Cd1—N1—C4	89.7 (3)	O4—Cd1—O2—N7	156.87 (19)
O2—Cd1—N1—C4	−61.7 (2)	O1—Cd1—O2—N7	−3.15 (17)
O1—Cd1—N1—C4	−111.3 (2)	O6—N8—O4—Cd1	−175.1 (2)
O7—Cd1—N1—C1	5.2 (3)	O5—N8—O4—Cd1	5.9 (3)
O8—Cd1—N1—C1	−157.6 (3)	O7—Cd1—O4—N8	−65.67 (19)
N4—Cd1—N1—C1	121.0 (3)	O8—Cd1—O4—N8	114.8 (2)
O4—Cd1—N1—C1	−83.3 (3)	N1—Cd1—O4—N8	31.1 (2)
O2—Cd1—N1—C1	125.4 (3)	N4—Cd1—O4—N8	−155.4 (2)
O1—Cd1—N1—C1	75.8 (3)	O2—Cd1—O4—N8	130.22 (19)
O7—Cd1—N4—C8	−32.6 (2)	O1—Cd1—O4—N8	−88.9 (3)
O8—Cd1—N4—C8	129.0 (2)	C2—N2—C1—N3	−178.9 (3)
N1—Cd1—N4—C8	−149.4 (3)	C2—N2—C1—N1	1.2 (5)
O4—Cd1—N4—C8	53.4 (2)	C4—N1—C1—N3	177.6 (3)
O2—Cd1—N4—C8	−153.7 (2)	Cd1—N1—C1—N3	−9.4 (4)
O1—Cd1—N4—C8	−104.5 (2)	C4—N1—C1—N2	−2.4 (5)
O7—Cd1—N4—C5	146.7 (3)	Cd1—N1—C1—N2	170.6 (2)
O8—Cd1—N4—C5	−51.7 (3)	C1—N2—C2—C3	0.9 (6)
N1—Cd1—N4—C5	29.8 (5)	N2—C2—C3—C4	−1.5 (6)
O4—Cd1—N4—C5	−127.3 (3)	C1—N1—C4—C3	1.7 (5)
O2—Cd1—N4—C5	25.6 (3)	Cd1—N1—C4—C3	−172.0 (3)
O1—Cd1—N4—C5	74.7 (3)	C2—C3—C4—N1	0.1 (6)
O3—N7—O1—Cd1	173.5 (3)	C6—N5—C5—N6	179.7 (4)
O2—N7—O1—Cd1	−5.5 (3)	C6—N5—C5—N4	−0.3 (5)
O7—Cd1—O1—N7	−178.1 (2)	C8—N4—C5—N5	1.3 (5)
O8—Cd1—O1—N7	−2.6 (2)	Cd1—N4—C5—N5	−177.9 (2)
N1—Cd1—O1—N7	81.30 (19)	C8—N4—C5—N6	−178.6 (3)
N4—Cd1—O1—N7	−87.48 (19)	Cd1—N4—C5—N6	2.1 (5)
O4—Cd1—O1—N7	−153.7 (2)	C5—N5—C6—C7	−1.0 (6)
O2—Cd1—O1—N7	3.13 (17)	N5—C6—C7—C8	1.0 (6)
O1—N7—O2—Cd1	5.8 (3)	C5—N4—C8—C7	−1.2 (5)
O3—N7—O2—Cd1	−173.2 (3)	Cd1—N4—C8—C7	178.1 (3)
O7—Cd1—O2—N7	−4.5 (2)	C6—C7—C8—N4	0.1 (6)

### Hydrogen-bond geometry (Å, °)

**Table:**

*D*—H···*A*	*D*—H	H···*A*	*D*···*A*	*D*—H···*A*
N3—H3A···O5^i^	0.86	2.29	3.105 (4)	158
N3—H3B···O7	0.86	2.10	2.945 (4)	167
N6—H6A···N5^ii^	0.86	2.20	3.054 (4)	170
N6—H6B···O2	0.86	2.19	2.931 (4)	144
N6—H6B···O3^iii^	0.86	2.52	3.171 (4)	133
O7—H7A···O9^iv^	0.85	1.94	2.787 (3)	178
O7—H7B···O9^v^	0.85	1.87	2.724 (3)	178
O8—H8A···O3^vi^	0.85	1.97	2.820 (3)	176
O8—H8B···O3^iii^	0.85	2.09	2.936 (3)	176
O9—H9A···O5^iv^	0.85	2.44	3.255 (3)	162
O9—H9A···O7^iv^	0.85	2.28	2.787 (3)	119
O9—H9B···O6^vii^	0.85	1.99	2.809 (4)	161

Symmetry codes: (i) −*x*, −*y*, −*z*; (ii) −*x*+1, −*y*, −*z*+1; (iii) −*x*+1, *y*+1/2, −*z*+1/2; (iv) −*x*, −*y*+1, −*z*+1; (v) *x*, −*y*+1/2, *z*−1/2; (vi) *x*, *y*+1, *z*; (vii) *x*, −*y*+3/2, *z*+1/2.
